# Time trends, uptake, and oncological effects of risk-reducing surgeries in 3067 Danish *BRCA1/2* carriers: a population-based study with matched controls

**DOI:** 10.1007/s10549-025-07821-4

**Published:** 2025-09-20

**Authors:** Cecilie Balslev Willert, Lene Mellemkjær, Anders Tolver, Anne-Marie Axø Gerdes, Susanne Rosthøj, Karin Wadt, Niels Kroman, Pernille Envold Bidstrup, Lisbet Rosenkrantz Hölmich

**Affiliations:** 1 Department of Plastic Surgery, Copenhagen University Hospital, Herlev and Gentofte, Borgmester Ib Juuls Vej 5, 2730 Herlev, Herlev, Denmark; 2 Diet, Cancer and Health, Danish Cancer Institute, Copenhagen, Denmark; 3 Statistics and Data Analysis, Danish Cancer Institute, Copenhagen, Denmark; 4 Department of Clinical Genetics, Rigshospitalet, Copenhagen University Hospital, Copenhagen, Denmark; 5 Department of Clinical Medicine, University of Copenhagen, Copenhagen, Denmark; 6 Danish Cancer Society, Copenhagen, Denmark; 7 Department of Breast Surgery, Copenhagen University Hospital, Herlev and Gentofte, Gentofte, Denmark; 8 Psychological Aspects of Cancer, Danish Cancer Institute, Copenhagen, Denmark

**Keywords:** *BRCA1/2* carriers, Risk-reducing bilateral mastectomy, Risk-reducing bilateral salpingo-oophorectomy, Breast cancer, Mortality

## Abstract

**Purpose:**

Knowledge of the uptake and breast and ovarian cancer-preventive and survival effects of bilateral risk-reducing mastectomy (BRRM) and salpingo-oophorectomy (RR-BSO) in female *BRCA1/2* carriers is essential for optimized decision-making. This study aimed to examine time trends in the number of registered unaffected *BRCA1/2* carriers, BRRM and RR-BSO uptake, and oncological effects of risk-reducing surgeries in a nationwide Danish cohort with matched controls.

**Methods:**

We included 3067 female *BRCA1/2* carriers registered in the Hereditary Breast and Ovarian Cancer Registry and 30,652 age-matched controls between 2000 and 2022. Data were retrieved from national health registries. Uptake and oncological effects of risk-reducing surgeries were assessed using cumulative incidences and Cox proportional hazards models with 95% confidence intervals (CI).

**Results:**

Annual numbers of registered unaffected *BRCA1/2* carriers, BRRM, and RR-BSO increased over time. BRRM and RR-BSO uptake 10 years after genetic test varied with the age at genetic test and parity. BRRM reduced the hazard rate of breast cancer by 94% [hazard ratio (HR) 0.06, CI 0.01–0.25]. The same pattern was not found for RR-BSO (HR = 1.31, CI 0.90–1.91). Compared to controls, *BRCA1/2* carriers had an increased hazard rate for breast cancer before BRRM (HR 7.49, CI 5.81–9.42).

**Conclusion:**

BRRM’s large protective effect against breast cancer in *BRCA1/2* carriers was confirmed, in contrast to that of RR-BSO. There were tendencies toward a reduction in overall mortality rates after BRRM, and compared with controls, we saw tendencies toward higher mortality rates before BRRM.

**Supplementary Information:**

The online version contains supplementary material available at 10.1007/s10549-025-07821-4.

## Introduction

While the penetrance varies by family history and specific gene variants, women with a pathogenic variant (PV) in the *BRCA1* or the *BRCA2* gene (*BRCA1/2* carriers) have an estimated cumulative lifetime risk of developing breast cancer of 50–72% [1–4]. In comparison, the cumulative risk for Danish females in the general population at age 80 is 11.5% [5].

Female *BRCA1/2* carriers have two preventive options regarding breast and ovarian cancer: a tailored surveillance program to detect potential breast and ovarian cancer as early as possible using radiological screening modalities and measurement with the biomarker CA-125 (for ovarian cancer) or surgical interventions with bilateral risk-reducing mastectomy (BRRM) and risk-reducing salpingo-oophorectomy (RR-BSO) [6]. The 2022 European Society of Medical Oncology (ESMO) guidelines state that BRRM is the most effective approach towards breast cancer risk reduction and should be considered individually, discussing the benefits and drawbacks with the patient [7]. Due to limited evidence of screening for ovarian cancer, ESMO guidelines recommend RR-BSO at ages 35–40 years for *BRCA1* carriers and 40–45 years for *BRCA2* carriers [7]. Both recommendations are in line with the current Danish Guidelines [6, 8]. An immediate breast reconstruction (IBR) is often performed simultaneously with BRRM, making this an extensive surgery with risks of surgical complications and permanent side effects, such as chronic pain in the chest area, as well as potential changes in body image, health-related quality of life and mental well-being [9–14]. Several studies have found that BRRM decreases the risk of breast cancer by 90–95% [15–18]. However, a Danish study from 2011 was unable to replicate this finding, likely due to its small sample size of 307 *BRCA1/2* carriers [19]. This underscores the need for an updated analysis of BRRM’s risk-reducing effect in a larger Danish cohort. Beyond lowering breast cancer incidence, BRRM has also been shown to improve overall survival in *BRCA1* carriers [20]. Although debated, RR-BSO may also reduce breast cancer risk, therefore, it is important to consider RR-BSO when estimating breast cancer risk in *BRCA1/2* carriers [21, 22]. It is well established that RR-BSO reduces overall mortality in women with a pathogenic variation in *BRCA1/2*, plausibly due to reduced number of ovarian cancers [23–25].

The increased use of genetic testing in breast and ovarian cancer patients has led to the identification of more relatives who are carriers of *BRCA1/2* pathogenic variants in need of genetic counseling and preventive strategies. Current Danish guidelines recommend genetic testing of all patients with ovarian cancer and breast cancer patients < 50 years to identify at-risk relatives and guide PARP (poly adenosine diphosphate-ribose polymerase) inhibitor treatment in ovarian cancer, as it is currently not approved for breast cancer in Denmark [6, 26, 27]. Additional genetic testing criteria also apply [6]. Despite the oncological benefits of BRRM, many women struggle with the decision of whether to undergo BRRM. Factors that increase the decision to have BRRM include higher parity, lower age at *BRCA1/2* carrier diagnosis, more breast cancer worry, and a history of more affected relatives [28–31].

Knowledge of the uptake and consequences of BRRM and RR-BSO is essential to optimize individualized preventive strategies and decision-making. With this study, we aimed to describe the incidence of newly identified unaffected female *BRCA1/2* carriers from 2000 to 2022, as well as the uptake of BRRM and RR-BSO. In addition, we aimed to estimate the risk of breast cancer and overall mortality in *BRCA1/2* carriers according to BRRM and RR-BSO status and compared to a matched control group. The central hypotheses were that BRRM reduces breast cancer rates and overall mortality and that RR-BSO reduces overall mortality but not breast cancer rates.

## Methods and materials

### Study design

This nationwide retrospective cohort study utilized data from seven national health registries. The study was approved by the Regional Danish General Data Protection Agency (ID P-2024-17747) and the Danish Cancer Society Research Center (record number 2019-DCRC-0097). As an observational study, it did not require approval from the Committee on Health Research Ethics.

The study adhered to the checklist for cohort studies from the Strengthening the Reporting of Observational Studies in Epidemiology (STROBE) Statement (Supplementary material, Appendix A) [32].

### Study population and data sources

We identified 3067 female carriers of a *BRCA1/2* class 4 or 5 PV registered in the Hereditary Breast and Ovarian Cancer (HBOC) Register, a part of the Danish Breast Cancer Group (DBCG) Register [33, 34], in the years 2000–2022 who, at their date of genetic test disclosure (the index date), were aged 18–80 years, alive, had Danish residency, had no history of cancer (except for non-melanoma skin cancer), no history of breast carcinoma in situ, and no previous mastectomy. Eight women who were both *BRCA1* and *BRCA2* carriers were classified as *BRCA1* carriers.

For each carrier, we randomly selected female controls from the background population matched 1:10 by age at the index date from the Danish Civil Registration System [35] using the same inclusion criteria as for BRCA carriers. From the 30,670 matched controls, 18 persons were excluded because they were identified as *BRCA1/2* carriers before their index date.

### Information on sociodemographic and reproductive factors

We retrieved information on the index day of age, vital status (dead, alive), and place of residency (Denmark, not Denmark) from the Danish Civil Registration System [35], education (short, medium, long) and cohabitant partner status (cohabitant, not cohabitant) from Statistics Denmark, and parity (0, 1, 2, > 2) and age in years at first childbirth including stillbirths (no child, ≤ 27, 28–29, ≥ 30 years) from the Danish Fertility Database, the Danish Civil Registration System, and the National Patient Registry [35–37].

### Information on risk-reducing surgeries, cancer, and death

We retrieved information on cancer diagnoses and dates from the Danish Cancer Registry [38] and breast carcinoma in situ diagnoses and dates from the Danish Pathology Register [39]. Information on patients’ history of risk-reducing surgeries (BRRM, yes/no, date, and RR-BSO, yes/no, date) and breast reconstruction (yes/no, date), as well as mastectomy type (skin-sparing, nipple-sparing, total, other) and breast reconstruction timing (immediate/delayed), was retrieved from the National Patient Register [37]. For cancer diagnoses < 90 days after bilateral mastectomy, the surgery was not considered risk-reducing. Thus, cancers discovered during BRRM were included in the non-BRRM group. Finally, information on causes of death (breast cancer, ovarian cancer, other cancer, non-cancerous) was obtained from the Danish Register of Causes of Death [40].

### Statistical analyses

Descriptive statistics were used to compare sociodemographic, reproductive, and cancer-related characteristics of *BRCA1/2* carriers, analyzed both combined and separately, and compared to the matched control population. Surgical characteristics were compared between the *BRCA1* and *BRCA2* carriers. For categorical variables, we applied either the Chi-square test or Fisher’s Exact Test, and for numeric variables, we used the Wilcoxon Rank Sum Test.

Women were followed until the occurrence of invasive breast cancer, death, emigration, or end of study (December 12, 2022), whichever came first. Median follow-up time was estimated using the reverse Kaplan–Meier estimator. The uptake of BRRM and RR-BSO, as well as breast cancer and mortality rates, were estimated with time-to-event analyses using time since *BRCA1/2* test disclosure for carriers or index date for matched controls as the underlying time-scale. Cumulative incidences of BRRM and RR-BSO were calculated using the Aalen–Johansen estimator [41], treating breast cancer and death as competing risks in analyses involving BRRM, ovarian cancer, and death as competing risks involving RR-BSO, and death as a competing risk in analyses of breast and ovarian cancer, respectively.

To analyze if the birth patterns differed between the carriers and controls, cumulative incidences of next childbirth (using death as competing event) among controls, *BRCA1* and *BRCA2* carriers were estimated as a function of time since genetic test disclosure (carriers) or index date (controls) stratified on age (18–29, > 30 years) and on parity at baseline (nulliparous, primiparous, multiparous).

To estimate the hazard ratio (HR) of breast cancer and overall mortality according to BRRM and RR-BSO status in *BRCA1/2* carriers, we applied Cox proportional hazards models incorporating a time-dependent variable with three states (no surgery, only RR-BSO, BRRM (including BRRM only and the combination of BRRM and RR-BSO because there were no events in the BRRM only group). The Cox models used age as timescale and were stratified for *BRCA1/2* status to allow different baseline hazards within each *BRCA* subtype and adjusted for parity (0, 1, 2, > 2) and age at first childbirth (≥ 30 or no child, 28–29, < 27 years) at index date. In a similar analysis, we compared the rate of breast cancer and death before and after BRRM for the *BRCA1/2* carriers to that of the matched control population.

Ninety-five percent confidence intervals (CIs) and *p* values were two-sided with a significance level of 0.05. All analyses were performed using R version 4.3.2 [32]. According to the Danish General Data Protection Regulation policy at the Danish Health Data Authority, counts with less than five observations are denoted “ < 5” [42]. Intervals are used to comply with the policy when necessary.

## Results

**Fig. 1:**
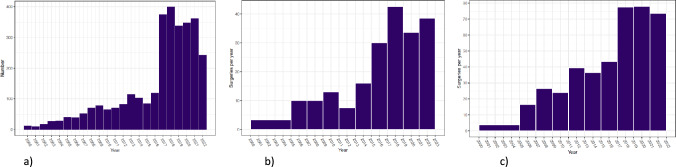
Annual numbers of (**a**) newly diagnosed unaffected female carriers of *BRCA1* and *BRCA2* pathogenic variants, (**b**) bilateral risk-reducing mastectomies, and (**c**) bilateral risk-reducing salpingo-oophorectomies from 2000 to 2022

We included 1649 *BRCA1* and 1418 *BRCA2* carriers (total *BRCA1/2* *n* = 3067) and 30,652 matched controls with median ages of 39 years and follow-up times from the index date of 4.5 years. Sixteen percent of the *BRCA1/2* carriers were below 25 years old (Table 1). Carriers and controls differed significantly in terms of level of education, cohabitant status, parity, and age at first childbirth. Comparing *BRCA1* with *BRCA2* carriers, differences were seen in median age, pathological class, level of education, year of birth, parity, and age at first childbirth (Table 1). Identification of *BRCA1/2* PV in unaffected carriers increased over time, with a substantial increase from 2017 (Fig. 1a).

**Table 1:** Sociodemographic and reproductive characteristics of 3067 female BRCA1/2 pathogenic variant carriers and 30,652 matched women in the control population

Characteristic	*BRCA1/2* carriers *N* (%)	Control population *N* (%)	*p* value^a^	*BRCA1* carriers *N* (%)	*BRCA2* carriers *N* (%)	*p* value^b^
*N*	3067	30,652	–	1649 (53.8)	1418 (46.2)	–
Age, median^c^	39.2	39.2	–	37.8	41.5	< 0.0001
Age intervals						
18–24 years	497 (16.2)	4982 (16.3)		305 (18.5)	192 (13.5)	
25–29 years	423 (13.8)	4204 (13.7)		244 (14.8)	179 (12.6)	
30–34 years	314 (10.2)	3153 (10.3)		169 (10.2)	145 (10.2)	
35–39 years	346 (11.3)	3444 (11.2)		196 (11.9)	150 (10.6)	
40–44 years	341 (11.1)	3406 (11.1)		175 (10.6)	166 (11.7)	
≥ 45 years	1146 (37.4)	11,463 (37.4)		560 (34.0)	586 (41.3)	< 0.0001
Follow-up period, median (IQR), years	4.54 (2.28–8.10)	4.53 (2.22–8.05)	–	4.71 (2.48–8.80)	4.30 (2.14–6.50)	–
Pathological class						
Class 4	109 (3.6)	–	–	75 (4.5)	34 (2.4)	
Class 5	2958 (96.4)			1574 (95.5)	1384 (97.6)	0.0016
Education^c, d^						
Short	510 (16.6)	5968 (19.5)		308 (18.7)	202 (14.2)	
Medium	1207 (39.4)	12,889 (42.0)		665 (40.3)	542 (38.2)	
Long	1335 (43.5)	11,434 (37.3)	< 0.0001	665 (40.3)	670 (47.2)	< 0.0001
Cohabitant partner status^c^						
Cohabitant	2103 (68.6)	19,981 (65.2)		1113 (67.5)	990 (69.8)	
Not cohabitant	964 (31.4)	10,587 (34.5)	< 0.0001	536 (32.5)	428 (30.2)	
Unknown	0	84 (0.3)		0	0	0.1723
Year of birth						
1939	17 (0.6)	170 (0.6)		12 (0.7)	5 (0.4)	
1940–1949	171 (5.6)	1710 (5.6)		80 (4.9)	91 (6.4)	
1950–1959	374 (12.2)	3738 (12.2)		189 (11.5)	185 (13.0)	
1960–1969	543 (17.7)	5427 (17.7)	–	295 (17.9)	248 (17.5)	
1970–1979	639 (20.8)	6386 (20.8)		324 (19.6)	315 (22.2)	
> 1980	1323 (43.1)	13,221 (43.1)		749 (45.4)	574 (40.5)	0.0160
Median	1976	1976		1978	1975	
Parity^c^						
0	1006 (32.8)	11,142 (36.3)		579 (35.1)	427 (30.1)	
1	421 (13.7)	4816 (15.7)		225 (13.6)	196 (13.8)	
2	1101 (35.9)	9640 (31.4)		569 (34.5)	532 (37.5)	
3 or more	539 (17.6)	5054 (16.5)	< 0.0001	276 (16.7)	263 (18.5)	0.0268
Age at first childbirth^c^, years						
No children	1006 (32.8)	11,142 (36.3)		579 (35.1)	427 (30.1)	
< 27	1283 (41.8)	12,659 (41.3)		704 (42.7)	579 (40.8)	
28–29	300 (9.8)	2567 (8.4)		161 (9.8)	139 (9.8)	
≥ 30	478 (15.6)	4284 (14.0)	< 0.0001	205 (12.4)	273 (19.3)	< 0.0001

^a^*p* value for comparison between controls and all *BRCA1/2* carriers

^b^*p* value for comparison between *BRCA1* and *BRCA2* carriers

^c^At the date of genetic test disclosure (index date)

^d^Missing data are not reported because some columns contain numbers < 5, which are not allowed to be reported according to the Danish General Data Protection Regulation policy at the Danish Health Data Authority

*IQR* inter-quartile range

### Risk-reducing surgeries

**Fig. 2:**
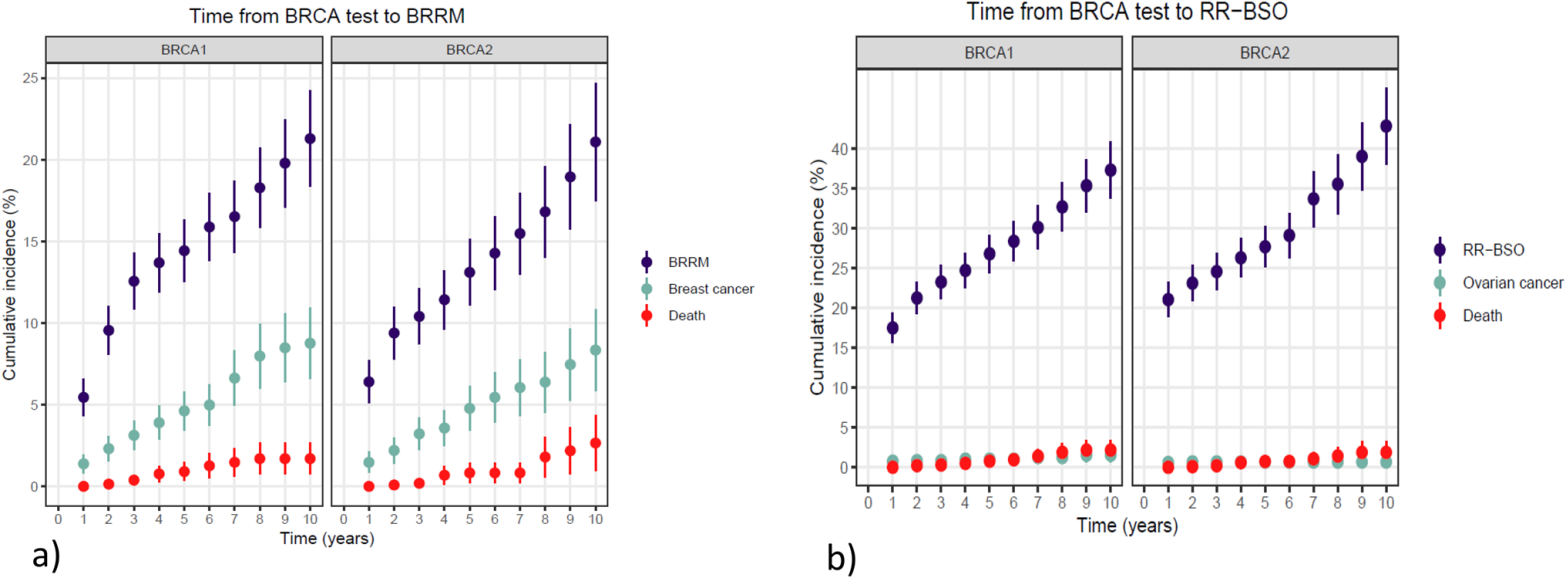
Cumulative incidences of bilateral risk-reducing mastectomy and bilateral salpingo-oophorectomy in unaffected female carriers of a *BRCA1* or *BRCA2* pathogenic variant as a function of time from genetic test disclosure in 1649 female *BRCA1* carriers and 1418 female *BRCA2* carriers. Breast cancer/ovarian cancer and death were considered competing events. BRRM: bilateral risk-reducing mastectomy; RR-BSO: risk-reducing bilateral salpingo-oophorectomy

During the study period, 419 (14%) and 850 (28%) of the 3067 *BRCA1/2* carriers underwent BRRM and RR-BSO, respectively. A subgroup of 296 women had both surgeries, of which most had BRRM after RR-BSO (Table 2). Ten years after genetic testing, the cumulative incidences of BRRM were 21% for both *BRCA1* (CI 18–24%) and *BRCA2* carriers (CI 18–25%) (Fig. 2a). The cumulative incidences of RR-BSO 10 years after genetic testing were 37% (CI 34–41%) for *BRCA1* carriers and 43% (CI 38–48%) for *BRCA2* carriers (Fig. 2b).

**Table 2:** Characteristics of risk-reducing surgeries and breast reconstruction among 3067 female *BRCA1/2* carriers

Characteristic	*BRCA1/2*	*BRCA1*	*BRCA2*	*p* value
*N*	3067	1649	1418	
Follow-up time from BRRM, median (IQR), years	5.3 (2.80–9.22)	6.10 (2.65–11.54)	4.82 (2.81–7.81)	–
Risk-reducing surgeries, *N* (%)^a^				
BRRM	419 (14.3)	240 (14.6)	179 (12.6)	
RR-BSO	850 (27.6)	543 (27.5)	395 (27.9)	
Combinations of surgeries, *N* (%)^a^				
None	2096 (68.3)	1125 (68.2)	969 (68.3)	
Only BRRM	123 (4.0)	71 (4.3)	52 (3.7)	
Only RR-BSO	554 (18.1)	284 (17.2)	270 (19.0)	
BRRM before RR-BSO	86 (2.8)	51 (3.1)	35 (2.5)	
BRRM after RR-BSO	210 (6.8)	118 (7.2)	92 (6.3)	0.4484
Mastectomy type				
Skin sparing	189 (45.1%)	104	85	
Nipple sparing	169 (40.3)	104	65	
Total	55 (13.1)	28–32	25–29	
Other and unknown	6 (1.4)	< 5	< 5	0.4925
Bilateral breast reconstruction				
No	34 (8.1)	20 (8.3)	14 (7.8)	
Immediate	366 (87.4)	208 (86.7)	158 (88.3)	0.8758
Secondary	19 (4.5)	12 (5.0)	7 (3.9)	

^a^Number (%) of individuals who underwent risk-reducing surgeries in this study within the observation period. These percentages should not be generalized to other studies or populations with different follow-up times

*IQR* inter-quartile range, *BRRM* bilateral risk-reducing mastectomy, *RR-BSO* risk-reducing bilateral salpingo-oophorectomy

The number of risk-reducing surgeries per year increased between 2000 and 2022 from < 5 to 37 for BRRM and < 5 to 43 for RR-BSO, with a steep increase in 2017 (Fig. 1b and c). The median follow-up time from BRRM to the end of the study was 5.3 years for *BRCA1/2* carriers. More than 90% of the *BRCA1/2* carriers who underwent BRRM had a breast reconstruction, of which > 94% were immediate. There were no differences in risk-reducing surgeries or breast reconstruction distributions between *BRCA1* and *BRCA2* carriers (Table 2). Twenty-three *BRCA1/2* carriers (0.1%) had both surgeries within 2 months (data not shown).

**Fig. 3:**
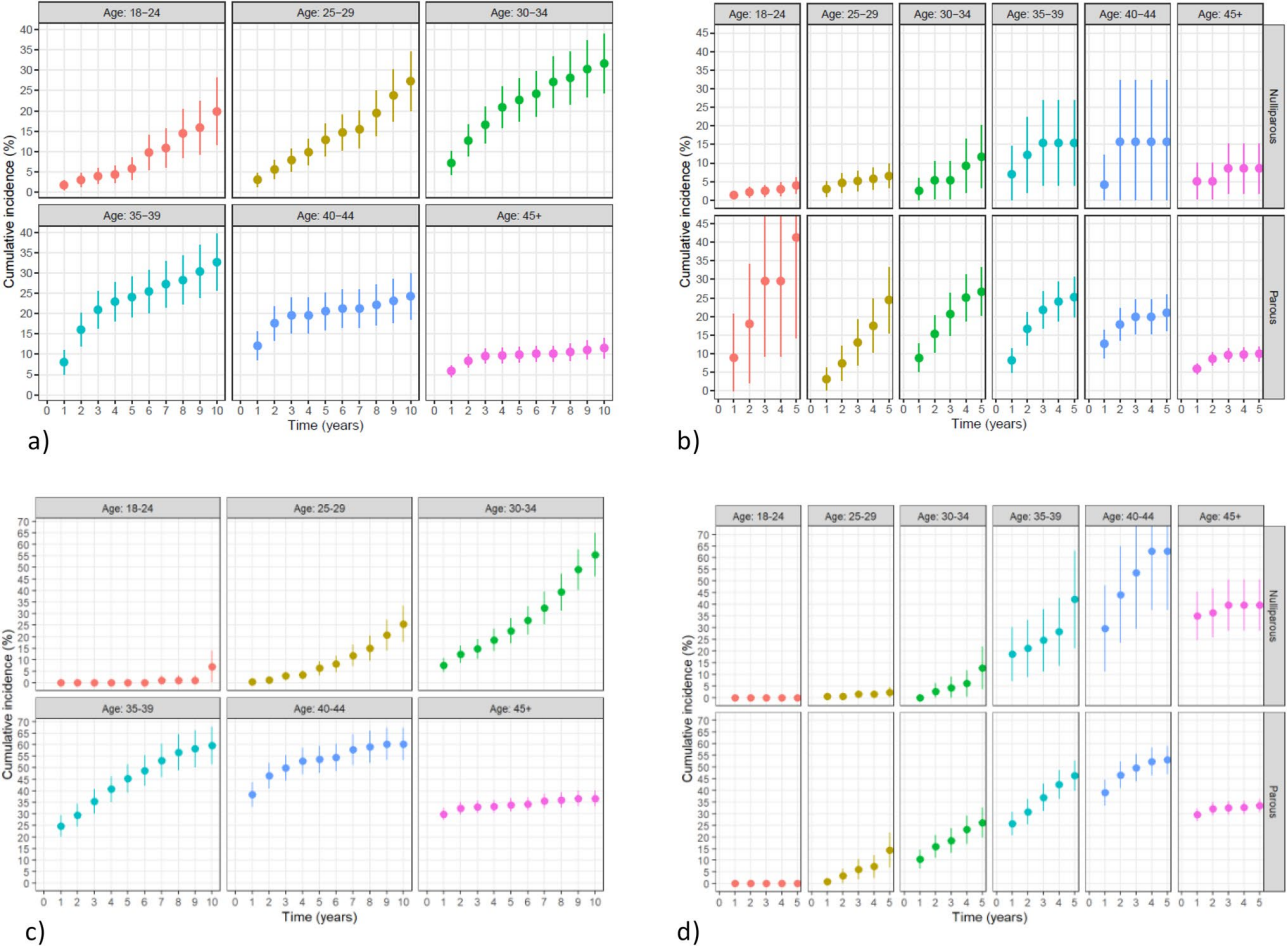
Cumulative incidences of bilateral risk-reducing mastectomy and bilateral salpingo-oophorectomy after genetic test disclosure in unaffected female *BRCA1* and *BRCA2* carriers stratified on (**a** and **c**) 5 years age intervals and (**b** and **d**) further stratified according to parity (nulliparous or parous). From time-to-event analyses with breast cancer and deaths as competing events in 1649 female *BRCA1* carriers and 1418 female *BRCA2* carriers

Uptake of BRRM 10 years after genetic test disclosure was highest for women aged 25–39 years (range 27–33%) (Fig. 3a). The corresponding RR-BSO uptake peaked at the ages 30–44 (range 56–60%) (Fig. 3c). Five years from the index date, the uptake of BRRM and RR-BSO was higher among parous *BRCA1/2* carriers than nulliparous at the ages 18–45 and 25–39 years, respectively (Fig. 3b and d). However, no difference was seen in terms of time between the index date and the next-born child compared to the matched control population (data not shown).

### Breast cancer risk

Fewer than five women were diagnosed with breast cancer in the BRRM group, 153 in the non-BRRM group, 100 in the RR-BSO group, 55 in the non-RR-BSO group, and 281 in the matched control population (Table 3). There were no carcinoma in situ events after BRRM (data not shown). Among *BRCA1/2* carriers, we found a statistically significant risk-reducing effect on breast cancer according to BRRM (with or without RR-BSO) (adjusted HR 0.06, CI 0.02–0.25) (Table 3), but not according to RR-BSO (adjusted HR 1.31, CI 0.90–1.91). Similar results were seen when analyzing *BRCA1* and *BRCA2* carriers separately (data not shown). Compared to the matched controls, *BRCA1/2* carriers had statistically significant increased breast cancer rates (adjusted HR 7.40, CI 5.81–9.42), but after BRRM, the rate did not differ (adjusted HR 0.47, CI 0.12–1.90) (Table 3).

**Table 3:** Hazard ratio (HRs) and 95% confidence intervals (CIs) for breast cancer and overall mortality according to risk-reducing surgeries among 3067 female *BRCA1/2* carriers and 30,652 matched controls

Breast cancer	Person-years at risk	Number of events^a^	Unadjusted HR (CI)	*p* value	Adjusted HR^b^ (CI)	*p* value
*BRCA1/2* carriers^c^						
Risk-reducing surgery status^d^						
No risk-reducing surgery	10,952	105	1.00		1.00	
BRRM ± RR-BSO	2834	< 5^e^	0.06 (0.01–0.24)	< 0.0001	0.06 (0.01–0.25)	< 0.0001
RR-BSO alone	3176	46	1.32 (0.91–1.91)	0.144	1.31 (0.90–1.91)	0.157
*BRCA1/2* carriers and the matched control population						
BRRM status^f^						
Matched controls	174,997	281	1.00		1.00	
BRCA 1/2 carriers with no BRRM	14,128	153	6.81 (5.59–8.29)	< 0.0001	7.40 (5.81–9.42)	< 0.0001
BRCA 1/2 carriers after BRRM	2834	< 5^e^	0.43 (0.11–1.75)	0.240	0.47 (0.12–1.90)	0.287

Overall mortality	Person-years at risk	Number of events^a^	Unadjusted HR (CI)	*p* value	Adjusted HR^ab^ (CI)	*p* value
*BRCA1/2* carriers^c^						
Risk-reducing surgery status^d^						
No risk-reducing surgery	11,262	36	1.00		1.00	
BRRM ± RR-BSO	2844	< 5^e^	0.35 (0.10–1.16)	0.087	0.34 (0.10–1.15)	0.083
RR-BSO alone	3773	15	0.91 (0.48–1.71)	0.235	0.93 (0.49–1.77)	0.830
*BRCA1/2* carriers and the matched control population						
BRRM status^f^						
Matched control	176,311	488	1.00		1.00	
BRCA 1/2 carrier with no BRRM	15,034	51	1.13 (0.84–1.51)	0.415	1.41 (0.99–2.02)	0.057
BRCA 1/2 carrier after BRRM	2844	< 5^e^	0.66 (0.21–2.07)	0.479	0.81 (0.26–2.59)	0.730

^a^Breast cancers diagnosed at BRRM are included in the group without BRRM (see the Methods and Materials section)

^b^Adjusted models further include parity at index date (0, 1, 2, > 2) and age at first childbirth (no child or > 30, < 27, 27–28 years) as covariates

^c^Models for *BRCA1/2* carriers are stratified to allow for different baseline hazards within each *BRCA* subtype

^d^Risk-reducing surgeries are included as time-dependent variables starting at the reference state “no surgery,” with possible transitions to “BRRM” (including the combination of BRRM and RR-BSO because there were no events in the BRRM group alone) or “only RR-BSO”

^e^According to the Danish General Data Protection Regulation policy at the Danish Health Data Authority, counts with less than five observations are denoted “ < 5” (https://sundhedsdatastyrelsen.dk/Media/638695056438446333/Hjemsendelse-af-analyseresultater.pdf)

^f^Models for *BRCA1/2* carriers and matched controls incorporated a time-dependent variable with two states (“before BRRM” and “after BRRM”) to compare the rate of breast cancer for *BRCA1/2* carriers before and after BRRM, with the matched non-carriers as reference

*BRRM* bilateral risk-reducing mastectomy, *RR-BSO* risk-reducing bilateral salpingo-oophorectomy

### Mortality risk

During the study period, less than five carriers died after BRRM (all from ovarian cancer or other malignancies), 51 carriers died without having had BRRM (most due to either breast, ovarian or other cancer), 15 carriers died after RR-BSO alone (most due to other cancers and none from ovarian cancer), 36 carriers died without having had any risk-reducing surgeries (most due to either breast, ovarian or other cancer), and 488 matched controls died (most due to non-cancerous causes) (Table 3). Causes of death are not shown. Mortality after BRRM (including *BRCA1/2* carriers who had both BRRM and RR-BSO performed) was reduced by 66%, although not reaching statistical significance (adjusted HR 0.34, CI 0.10–1.15). RR-BSO did not statistically significantly reduce mortality (adjusted HR 0.93, CI 0.49–1.77). Finally, compared with the matched controls, *BRCA1/2* carriers had increased mortality rates before BRRM (adjusted HR 1.41, CI 0.99–2.02), although only borderline statistically significant (Table 3).

## Discussion

To our knowledge, this is the first study to compare unaffected *BRCA1/2* carriers with matched controls regarding sociodemographic and reproductive characteristics, as well as breast cancer and mortality rates. BRRM and RR-BSO uptakes ten years after genetic test were 21% and approximately 40%, respectively, but depended on age and parity. BRRM reduced breast cancer incidence as hypothesized, and a survival benefit was indicated, while we were unable to find a difference in breast cancer and mortality rates after RR-BSO.

The number of registered unaffected *BRCA1/2* carriers and the annual number of risk-reducing surgeries increased over time during the 22-year-long study period, reflecting an increased awareness of genetic predisposition for breast and ovarian cancer in both unaffected and affected women, as well as lowered costs of genetic testing [43]. There was a notable rise in genetic testing and risk-reducing surgeries in 2013 and particularly in 2017. This is likely attributable to the recommendation to test all patients with epithelial ovarian cancer implemented in 2017, leading to the identification of more unaffected relatives in need of risk-reducing surgeries. The overall increase in genetic testing is in line with recently reported increasing test rates of Danish breast cancer patients from 2000 to 2017 [44]. The increasing numbers in 2013–2016 may be related to celebrity Angelina Jolie’s announcement that she was a *BRCA1* carrier and had undergone BRRM in 2013 [45, 46].

*BRCA1/2* carriers were more likely to have a cohabitant partner and be parous with a higher level of education than the matched controls. Perhaps women with higher levels of education are more likely to seek genetic testing, as has been reported in the USA [47]. Akin to previous studies, our results indicate that the uptake of risk-reducing surgeries is higher among parous women compared with nulliparous [28, 48].

Compared with the Danish 2010 cohort, our BRRM uptake after 10 years was substantially lower (21% versus 50%) [48]. This may be due to the 2007 launch of a protocol with magnetic resonance imaging scans in addition to mammography and ultrasound, which may have caused more women to select surveillance over surgery. This option was only available in the final two years of their observation period [48, 49]. Similarly, RR-BSO uptake 10 years after the genetic test of 37% and 43% for *BRCA1* and *BRCA2* carriers, respectively, was much lower compared with the reported 10-year uptake in the Danish study from 2010 (75%) [48]. Importantly, the cumulative incidences, and thus the generalizability, strongly depend on the distribution of age at time of DNA test, as is illustrated in Fig. 3.

Many previous studies reporting BRRM uptake have not considered follow-up time and competing events in the analyses [50]. A time-to-event analysis of 479 unaffected British *BRCA1/2* carriers with up to 32 years of follow-up found a BRRM uptake of 48% 10 years after genetic testing [28]. Similarly, a study from the USA of 1499 *BRCA1/2* carriers found a BRRM uptake of 46% at age 60 [50]. The large differences from our BRRM and RR-BSO uptakes 10 years after genetic testing may be due to differences in follow-up time, number and age of participants, competing risks, clinical guidelines, culture, clinician attitudes, and healthcare insurance systems [28, 51]. For most of our study period, Danish *BRCA1/2* carriers have been recommended a tailored radiological imaging surveillance program due to a lack of evidence of a survival benefit from BRRM. This means that the BRRM group actively sought surgery. This approach has changed in recent years, as patients are now informed of evidence of a suggested survival benefit to BRRM for *BRCA1* carriers [20, 52], which may cause the BRRM uptake to increase in the years to come.

Our results confirm the previously established breast cancer risk-reducing effect of BRRM [15–17] and suggest that the lack of statistical significance in the Danish study from 2010 was likely due to its small sample size of 307 *BRCA1/2* carriers [19]. We did not find an effect of RR-BSO on breast cancer incidence in *BRCA1/2* carriers, neither combined nor separately. RR-BSO has previously been found to reduce the risk of breast cancer in female *BRCA1/2* carriers by 50% [22], but the effect was questioned in other studies [21]. A more recent systematic review and meta-analysis found that the benefit may be restricted to *BRCA2* carriers [53]. Our lack of significance may be ascribed to the short follow-up time of 4.5 years and the median age of 39 years. Adjustment for reproductive factors did not change the estimates, suggesting that these breast cancer risk factors have negligible effects as confounders for the association between BRRM and RR-BSO and breast cancer in *BRCA1/2* carriers.

Compared with the matched controls, the breast cancer rate was reduced by a factor of 0.47 (CI 0.12–1.90) after BRRM in the 419 operated *BRCA1/2* carriers. Our lack of statistical significance is likely ascribed to our limited number of women followed post-BRRM.

Overall mortality ratios after BRRM in *BRCA1/2* carriers were comparable to those observed for *BRCA1* carriers in the Dutch study (adjusted HR 0.34 versus 0.40) [20]. Although similar in sample size, our study had a shorter follow-up, which may explain why our results did not reach statistical significance. Similarly, a study of 1654 *BRCA1/2* carriers failed to demonstrate statistically significant reduced breast cancer mortality rates after BRRM (HR 0.26, CI 0.05–1.35) [18]. As no breast cancer deaths occurred following BRRM in our study, we were unable to assess breast cancer-specific mortality. This suggests that the potential reduction in overall mortality is due to fewer breast cancer deaths. Interestingly, we observed a 41% higher overall mortality HR among *BRCA1/2* carriers before BRRM compared to matched controls, although not reaching statistical significance. This suggests that *BRCA1/2* carriers have higher mortality than the general population and emphasizes the importance of identifying unaffected carriers to initiate risk management strategies.

In contrast to previous studies, we did not find a reduction in overall mortality according to RR-BSO (adjusted HR 0.93, CI 0.49–1.77) [23–25], although no carriers died of ovarian cancer after RR-BSO. However, with a short follow-up time and a median age below the typical debut age for ovarian cancer in *BRCA* carriers [54] this is not surprising and cannot be interpreted as implying that RR-BSO does not have a survival benefit. In the recent study by Kotsopoulos et al., the women were followed from age 35 years [25]. In our cohort, 40% were < 35 years old, and the studies are therefore not comparable. In addition, we estimated the HR for carriers undergoing RR-BSO alone without accounting for those who had both surgeries. In future studies, this would be relevant.

### Limitations and strengths

Limitations include the fact that our study population of unaffected *BRCA1/2* carriers does not represent the population of all Danish carriers, as this would require genetic testing of the entire population. Estimates of BRRM and RR-BSO uptakes, as well as cumulative incidences of breast cancer and death as functions of age, generalize to all *BRCA1/2* carriers under certain untestable conditions regarding associations between age at *BRCA*-test disclosure (study entry) and time-to-event (surgery/breast cancer/death). To support our interpretation that the results can be generalized to the entire *BRCA1/2* population, we highlight two opposing sources of bias: (1) excluding *BRCA1/2* carriers tested after a breast cancer diagnosis may lead to an underestimation of breast cancer risk and (2) *BRCA1/2* carriers with lower penetrance—who may never develop or may develop breast cancer later in life—are less likely to be referred for genetic testing, which could lead to an overestimation of breast cancer risk. Also, we had a short follow-up time from the index date and BRRM (median 4.5 and 5.3 years, respectively) for *BRCA1/2* carriers, which decreased the precision of the estimates.

A key strength of our study is the high number of *BRCA1/2* carriers, approximately 10 times higher than in the previous Danish *BRCA1/2* cohort and considerably larger compared with other studies that examine the effects of BRRM on breast cancer incidence in *BRCA1/2* cohorts [17, 19, 48]. This increases the likelihood of events of interest. In addition, all of our information stem from the national health registries and is thus prospectively collected, population based, and nationwide. These sources of information ensure complete follow-up and minimize bias in assessing outcomes.

### Clinical perspectives

In future studies, it would be relevant to investigate if there are sub-groups that may benefit particularly from BRRM compared to others. The number of affected first-degree relatives and the specific locations of genetic variants may act as such effect modifiers [1]. In addition, it could be relevant to investigate sub-groups regarding known breast cancer risk modifiers, such as BMI, physical activity, alcohol consumption, hormonal contraceptives, menopausal hormone therapy, breast tissue density, breastfeeding, age at menarche, age at menopause [55], reproductive factors [56–58], breastfeeding [59], and physical activity [60, 61]. This knowledge would contribute to more individualized decision-making.

## Conclusion

This nationwide cohort study provides updated insights into the incidence of unaffected *BRCA1/2* carriers, BRRM and RR-BSO uptake, and their impact on breast cancer risk and mortality in Denmark, also compared with the general population. The numbers of newly diagnosed female unaffected *BRCA1/2* carriers and risk-reducing surgeries increased over the study period. Our findings confirmed BRRM’s significant risk-reducing effect by a factor of 0.06, while RR-BSO showed no impact on breast cancer incidence. Mortality rates for *BRCA1/2* carriers before BRRM were increased compared with the general population, while mortality following BRRM was reduced. In addition, our results indicate a possible survival benefit of BRRM. These results emphasize the importance of continued evidence-based genetic counseling for *BRCA1/2* carriers.

## Supplementary Information

Below is the link to the electronic supplementary material.

Supplementary file1 (DOCX 33 KB)

## Data Availability

The datasets generated and analyzed during the current study are not publicly available due to Danish law.
